# Supplementary material for: Active site specificity profiling datasets of matrix metalloproteinases (MMPs) 1, 2, 3, 7, 8, 9, 12, 13 and 14
Source: Data Brief. 2016 Feb 22;7:299–310. doi: 10.1016/j.dib.2016.02.036 (PMC4777984; doi:10.1016/j.dib.2016.02.036)
Supplement: Supplementary file 10 — Supplementary material [file mmc10.zip › WebPICS_hMMP9_G_1%/P1.html]

 

PICS results


|  |  |
| --- | --- |
| **P1\_A**  24 in 146 sites   16.4 %    effects > 10 perc. pnts.  (vice-versa in brackets)  P3\_A: 14.1 (15.4)   P2\_K: 11.3 (13.6)   P1prime\_Q: 21.0 (41.9)   P2prime\_K: 16.9 (22.5) |  |
| **P1\_C**  2 in 146 sites   1.4 %    effects > 10 perc. pnts.  (vice-versa in brackets)  P2prime\_Q: 45.9 (15.3) |  |
| **P1\_D**  14 in 146 sites   9.6 %    effects > 10 perc. pnts.  (vice-versa in brackets)  P1prime\_C: 13.9 (17.7) |  |
| **P1\_G**  21 in 146 sites   14.4 %    effects > 10 perc. pnts.  (vice-versa in brackets)  P1prime\_C: 11.5 (22.0)   P1prime\_F: 13.5 (35.6)   P2prime\_A: 14.9 (24.1)   P2prime\_T: 17.6 (41.2)   P3prime\_S: 21.7 (14.6) |  |
| **P1\_H**  5 in 146 sites   3.4 %    effects > 10 perc. pnts.  (vice-versa in brackets)  P2\_Y: 16.6 (16.6)   P2prime\_Q: 35.9 (29.9) |  |
| **P1\_K**  11 in 146 sites   7.5 %    effects > 10 perc. pnts.  (vice-versa in brackets)  P1prime\_I: 21.3 (10.7)   P3prime\_G: 33.3 (11.9) |  |
| **P1\_N**  12 in 146 sites   8.2 %    effects > 10 perc. pnts.  (vice-versa in brackets)  P1prime\_F: 11.2 (16.8)   P2prime\_I: 16.1 (14.9)   P3prime\_N: 20.9 (41.8) |  |
| **P1\_Q**  6 in 146 sites   4.1 %    effects > 10 perc. pnts.  (vice-versa in brackets)  P2\_Q: 12.6 (12.6)   P3prime\_N: 12.6 (12.6) |  |
| **P1\_R**  15 in 146 sites   10.3 %    effects > 10 perc. pnts.  (vice-versa in brackets)  P2\_K: 19.6 (14.7)   P2prime\_V: 26.3 (19.7) |  |
| **P1\_S**  16 in 146 sites   11.0 %    effects > 10 perc. pnts.  (vice-versa in brackets)  P2prime\_L: 12.7 (11.2) |  |
